# TFPI2 suppresses breast cancer progression through inhibiting TWIST-integrin α5 pathway

**DOI:** 10.1186/s10020-020-00158-2

**Published:** 2020-04-05

**Authors:** Danyi Zhao, Jingjing Qiao, Hongmei He, Jincheng Song, Shanshan Zhao, Jing Yu

**Affiliations:** Department of Oncology, The Second Affiliated Hospital of Dalian Medical University, No.467 Zhongshan Road, Shahekou District, Dalian City, 116027 Liaoning Province China

**Keywords:** TFPI2, TWIST1, Integrin α5, Breast cancer, Progression

## Abstract

**Background:**

Tissue factor pathway inhibitor 2 (TFPI2) participates in carcinogenesis of various tumors, and is associated with poor survival of breast cancer patients. However, the effect and underlying mechanism of TFPI2 on breast cancer progression remains to be investigated.

**Methods:**

The expression level of TFPI2 in breast cancer tissues and cell lines was examined via qRT-PCR (quantitative real-time polymerase chain reaction) and immunohistochemistry. CCK8 (Cell Counting Kit-8), colony formation, wound healing or transwell assays were used to detect cell viability, proliferation, migration or invasion, respectively. In vivo subcutaneous xenotransplanted tumor model was established to detect tumorigenic function of TFPI2, and the underlying mechanism was evaluated by immunohistochemistry and western blot.

**Results:**

TFPI2 was down-regulated in breast cancer tissues and cell lines, and was associated with poor prognosis of patients diagnosed with breast cancer. Over-expression of TFPI2 inhibited cell viability, proliferation, migration and invasion of breast cancer cells. Mechanistically, Twist-related protein 1 (TWIST1) was negatively associated with TFPI2 in breast cancer patients, whose expression was decreased by TFPI2 over-expression or increased by TFPI2 knockdown. Moreover, TWIST1 could up-regulate integrin α5 expression. Functional assays indicated that the inhibition abilities of TFPI2 over-expression on breast cancer progression were reversed by TWIST1 over-expression. In vivo subcutaneous xenotransplanted tumor model also revealed that over-expression of TFPI2 could suppress breast tumor growth via down-regulation of TWIST1-mediated integrin α5 expression.

**Conclusions:**

TFPI2 suppressed breast cancer progression through inhibiting TWIST-integrin α5 pathway, providing a new potential therapeutic target for breast cancer treatment.

## Background

Breast cancer is the most common and lethal malignant tumor among women worldwide, accounting for approximately 22% of all malignancies in women (Hashim et al. 2016). The incidence of breast cancer has increased nearly 10-fold over the past 10 years (Siegel et al. 2016). At present, surgical resection is still the main treatment for breast cancer patients in early stage accompanied by adjuvant chemotherapy (Lee and Chu 2018; Feng et al. 2018). Identification of new sensitive diagnosis biomarkers is crucial for the efficacy of chemotherapy for breast cancer. However, due to migration and invasion of breast cancer cells, metastasis becomes the main reason for treatment failure (Benson et al. 2009). Therefore, although comprehensive treatment modes have gradually reduced the death rate of breast cancer patients (Albuquerque et al. 2018), there is still a considerable necessary to continuously explore the molecular mechanism of breast cancer occurrence and metastasis and search for potential intervention targets.

Tissue factor pathway inhibitor 2 (TFPI2) is a proteolytic enzyme inhibitor with a Kunitz domain, and belongs to the superfamily of serine protease inhibitors (Chand et al. 2004). TFPI2 could inhibit extracellular matrix hydrolysis via suppression of matrix metalloproteinases (Herman et al. 2001). Recently, studies have shown that TFPI2 is down-regulated in prostate cancer (Konduri et al. 2001), pancreatic ductal adenocarcinoma (Sato et al. 2005), gastric cancer (Takada et al. 2010), non-small-cell lung cancer (Rollin et al. 2005), etc., and is closely related to various malignant tumors progression. Down-regulation of TFPI2 was found to be associated with poor survival in patients with breast cancer (Xu et al. 2013). CpG methylation in the promoter of TFPI2 inhibits binding ability between Kruppel-like factor 6 (KLF6) and TFPI2 in breast cancer (Guo et al. 2007). However, the effect and mechanism involved in the regulation of TFPI2 on breast cancer have not been reported.

TWIST1 belongs to basic helix-loop-helix transcription factor family, and functions as promoter of epithelial-to-mesenchymal transition, cell migration and invasion in cancer cells (Khan et al. 2013). TWIST1 promotes breast cancer cell migration and invasion via different signaling pathways (Xu et al. 2017a; Glackin 2014). Therefore, we hypothesized that the potential regulatory mechanism of TFPI2 on breast cancer dependents on TWIST1. We first investigated the expression level of TFPI2 in breast cancer, and the down-regulation of TFPI2 was regarded as the starting point to further discuss the function and mechanism of TFPI2 in the occurrence and development of breast cancer.

## Methods

### Patient samples and immunohistochemistry

Fifty surgical cancer specimens from breast cancer patients and 16 normal breast tissues were collected at The Second Affiliated Hospital of Dalian Medical University. The study was approved by the Ethics Committee of The Second Affiliated Hospital of Dalian Medical University, and all the patients signed written informed consent. Paraffin-embedded breast cancer tissues were cut into 5 μm thick sections. The sections were then dewaxed and rehydrated following by antigens retrieval and endogenous peroxidase blocking. After blocking with 2% goat serum in PBS, sections were then incubated overnight with primary rabbit antibodies against TFPI2 and TWIST1 (Abcam, Cambridge, MA, USA). HRP (horseradish peroxidase, Sigma Aldrich, St. Louis, MO, USA)-conjugated goat anti-rabbit IgG secondary antibody was then added, and the slides were counterstained with hematoxylin and examined under light microscope (Olympus, Tokyo, Japan).

### Cell culture

Human breast cancer cell lines (MDA-MB-453, MDA-MB-468 and MCF7), breast fibroblast cell line (CCD-1095Sk) and non-tumorigenic epithelial cell line (MCF10A) were purchased from Chinese Academy of Sciences (Shanghai, China), and cultured in Ham’s F12K (Gibco; Thermo Fisher, Waltham, MA, USA) supplemented with 10% fetal bovine serum at 37 °C constant temperature incubator with 5% CO_2_.

### Cell transfection

pcDNA3.1-TFPI2, pcDNA3.1-TWIST1 and the negative control (pcDNA3.1-NC) were obtained from AxyBio co., LTD (Changsha, China). shRNAs targeting TFPI2 (shTFPI2#1, #2, #3) or shTWIST1 and the negative control (shNC) were synthesized by GenePharma (Shanghai, China). 1 × 10^6^ cells/well of MDA-MB-453, MCF7 or MCF10A cells were seeded into 12-well plate and then transfected with pcDNA3.1-TFPI2, pcDNA3.1-TWIST1 andpcDNA3.1-NC or shTFPI2#1, #2, #3 and shNC via Lipofectamine® 3000 (Thermo Fisher, Waltham, MA, USA). Two days after transfection, the cells were collected for the following experiments.

### Cell proliferation assay

5 × 10^3^ cells/well MDA-MB-453 or MCF10A cells were seeded in 96-well plate. At 0, 1, 2, 4, 6 days, 20 μL CCK8 solution (Dojindo, Tokyo, Japan) was added into each well and mixed for 3 h. Microplate Autoreader (BioTek, Winooski, VT, USA) was used to measure optical density at 450 nm. For colony formation experiments, MDA-MB-453 or MCF10A cells were seeded on six-well plate with 200 cells/well and cultured with Ham’s F12K. Fourteen days later, the cells were fixed in formalin and stained with crystal violet (0.1%). The visible colonies were counted and photographed under light microscope.

### Wound healing and Transwell assay

For cell migration analysis, MDA-MB-453 or MCF10A cells were seeded in 6-well plates. Wound gap in the cell monolayer was generated by scratching with plastic pipette tip. The cells were washed with PBS to remove debris or the detached cells, and then cells were cultured in Ham’s F12K for another 48 h before calculating the wound width. For cell invasion analysis, MDA-MB-453 cells were seeded into the upper wells of chamber (Corning, MA, USA) with the Matrigel-coated membrane (BD Biosciences, Franklin Lakes, NJ, USA) in serum-free Ham’s F12K. Ham’s F12K with 10% FBS were added to the lower wells. The medium of upper wells and the filters were removed 8 h later. The invasive cells to the bottom of chambers were fixed with 100% methanol and then stained with 0.1% crystal violet 24 h later, imaged and counted under microscope.

### Quantitative real-time PCR (qRT-PCR)

Total RNAs from normal breast tissues, breast cancer tissues or cell lines were extracted via RNeasy Mini Kit (Qiagen, Manchester, UK). Complementary DNAs were then generated by PrimeScript RT Reagent (Takara, Shiga, Japan). qRT-PCR was analyzed by ViiA 7 (Applied Biosystems, Austin, TX, USA), and the relative expression of indicated genes were normalized to GAPDH and calculated using 2^-△△Ct^ methods. The primer sequences were listed in primer Table 1.

**Table 1:** Primer sequences

ID	Sequence(5′- 3′)
GAPDH F	ACCACAGTCCATGCCATCAC
GAPDH R	TCCACCACCCTGTTGCTGTA
TFPI2 F	GGGTTTATGGTGTAGGGGG
TFPI2 R	CAACCACCCCTCAAACTCC

### Western blot

30 μg proteins extracted from normal breast tissues, breast cancer tissues or cell lines were separated by sodium dodecyl sulfate-polyacrylamide gelelectrophoresis, and then transferred to nitrocellulose membrane (Millipore, Bedford, MA). The membranes were blocked by 5% skimmed milk and then incubated overnight with primary antibodies, including anti-TFPI2 antibodies (1:1500, Abcam), TWIST (1:2000, Abcam), integrin α5 (1:2500, Abcam), GAPDH (1:3000, Abcam) at 4 °C. Lastly, the immunoreactivities were detected by enhanced chemiluminescence (KeyGen, Nanjin, China) after incubating with HRP labeled secondary antibody (1:5000; Abcam).

### Mouse xenograft assay

Animal experiments were approved by the Ethics Committee of The Second Affiliated Hospital of Dalian Medical University. Twelve-seven weeks old female BALB/c nude mice were used and conducted in accordance with the National Institutes of Health Laboratory Animal Care and Use Guidelines. Ad-pcDNA 3.1-TFPI2 and the negative control (Ad-pcDNA 3.1-NC), were constructed by GenePharma (Shanghai, China). 100 μL of 2.5 × 10^6^ MDA-MB-453 cells transfected with Ad-pcDNA 3.1-TFPI2 or Ad-pcDNA 3.1-NC were injected into mice mammary fat pad. Tumors were measured and the volume was calculated at 7, 10, 14, 17, 21, 24 days. Twenty- four days later, mice were anesthetized with sodium pentobarbital at 65 mg per kg body weight, and the breast cancer tissues were collected for analysis.

### Statistical analysis

The data were shown as mean ± standard deviation, and the statistics analysis was performed by the SPSS 19.0 (SPSS, Chicago, IL). Student’s t text was used to compare the difference between two groups, one-way ANOVA with Turkey’s test to compare the difference among multiple groups. *p* < 0.05 was considered as statistically significant.

## Results

### TFPI2 was down-regulated in breast cancer tissues and cell lines

**Fig. 1:**
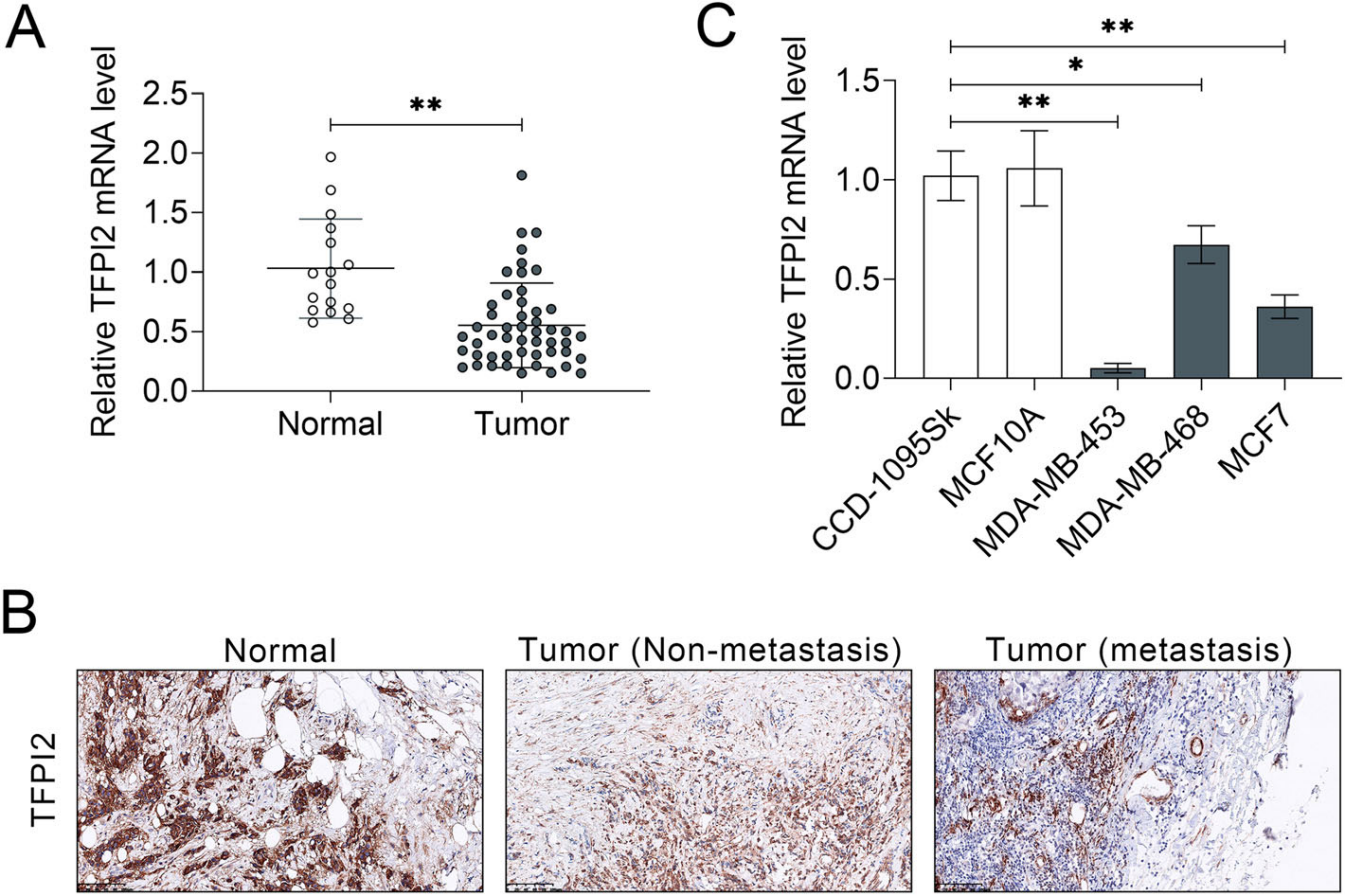
TFPI2 was down-regulated in breast cancer tissues and cell lines. **a** The expression of TFPI2 was down-regulated in breast cancer (Tumor) compared to normal breast specimens (Normal). ** represents Tumor vs. Normal tissues, *P* < 0.01. **b** Immunohistochemistry of TFPI2 in Normal, Non-metastasis or metastasis breast cancer tissues, suggesting down-regulation of TFPI2 in breast cancer tissues. **c** The expression of TFPI2 was down-regulated in human breast cancer cell lines (MDA-MB-453, MDA-MB-468 and MCF7) compared to CCD-1095Sk and MCF 10A. *, ** represents breast cancer cell lines vs. CCD-1095Sk, *P* < 0.05, *P* < 0.01

The expression of TFPI2 was first investigated in breast cancer tissues and cell lines. The results showed that the expression of TFPI2 in breast cancer was lower than in normal breast specimens (Fig. 1a). In addition, immunohistochemistry also showed that TFPI2 expression was negatively correlated to metastatic property of breast cancer. The results demonstrated thatmetastatic breast cancer tissues had the lowest expression of TFPI2, while non-metastatic breast cancer tissues showed higher and normal breast tissues presented the highest expression of TFPI2 (Fig. 1b). Therefore, TFPI2 may contribute to breast cancer progression. Further refinement analysis of correlation between TFPI2 and clinic pathologic characteristics of breast cancer patients indicated that among the 50 patients, low expression of TFPI2 (*N* = 25) was significantly related to tumor size (*P* = 0.005), metastasis (*P* = 0.018) and pathological stage (*P* = 0.010) (Table 2), suggesting the potential of TFPI2 as a prognostic biomarker for breast cancer. However, age (*P* = 0.777) and nodal status (*P* = 0.156) showed no significant correlation with TTFPI2 expression (Table 2). Consistent with expression in breast cancer tissues, TFPI2 was also down-regulated in human breast cancer cell lines (MDA-MB-453, MDA-MB-468 and MCF7) compared to breast fibroblast cell line (CCD-1095Sk) and MCF10A (Fig. 1c). MDA-MB-453 cell with lowest expression of TFPI2 was selected for the following gain-of functional assays, and MCF7 was selected for loss-of functional assay.

**Table 2:** Relationship between TFPI2 mRNA and clinico-pathological parameters

Parameters	Number of patients	TFPI2 mRNA expression		*P* value
		Low (< median)	High (≥ median)	
Number	50	25	25	
Age				
< Mean (47)	27	14	13	0.777
≥ Mean (47)	23	11	12	
Tumor size				
T1–2	26	18	8	0.005*
T3–4	24	7	17	
Nodal status				
N0–1	23	14	9	0.156
N2–3	27	11	16	
Metastasis				
M0	32	20	12	0.018*
M1	18	5	13	
Pathological stage				
I-II	29	19	10	0.010*
III-IV	21	6	15	

*represents a significant relation between TFPI2 and clinico-pathological parameters of breast cancer patients

### Over-expression of TFPI2 suppressed cell viability, proliferation, migration and invasion of breast cancer

**Fig. 2:**
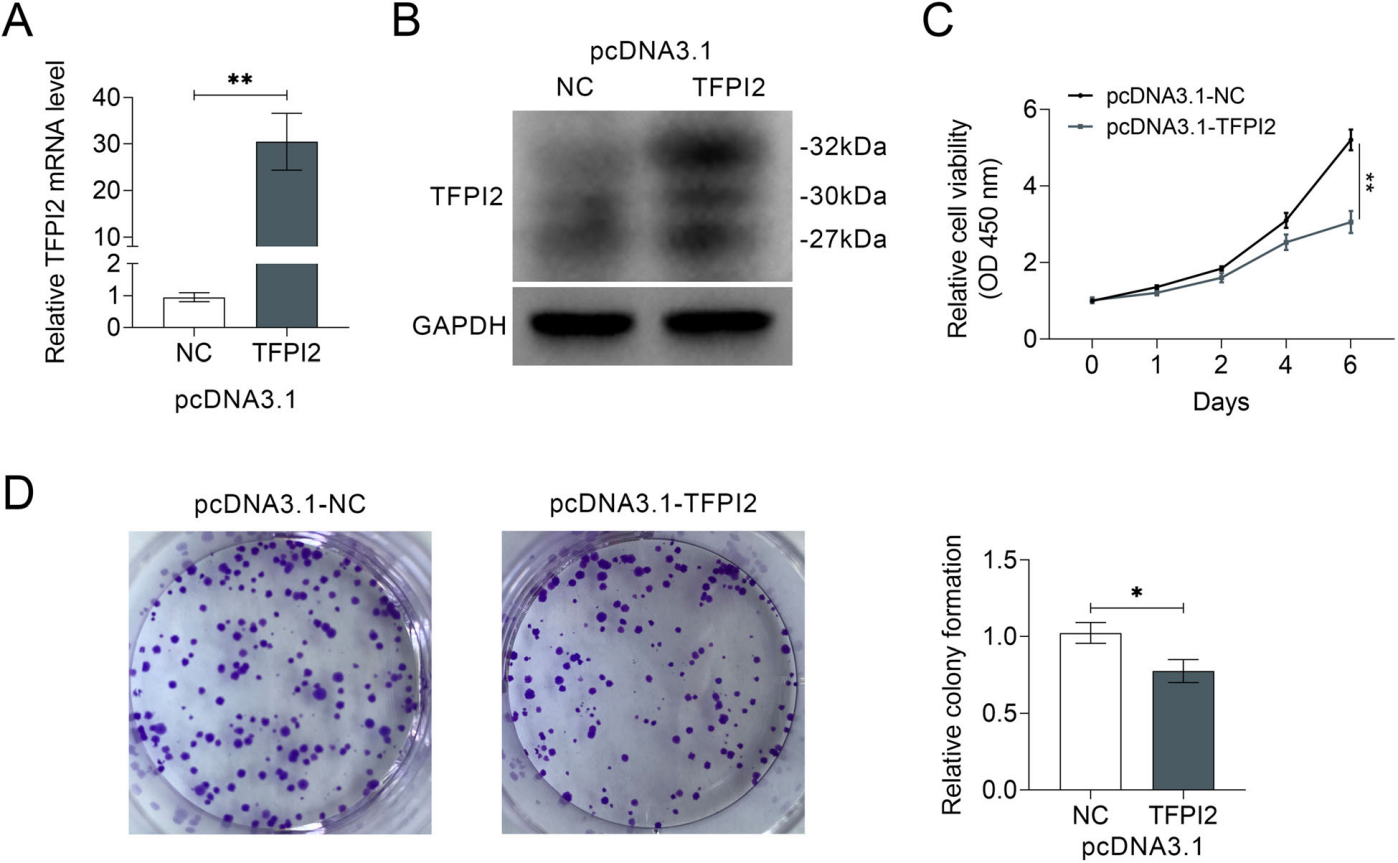
Over-expression of TFPI2 suppressed cell viability and proliferation of breast cancer. **a** Up-regulation of TFPI2 in MDA-MB-453 cells transfected with pcDNA 3.1-TFPI2 indicated by qRT-PCR. ** represents pcDNA 3.1-TFPI2 (TFPI2) vs. pcDNA 3.1-NC (NC), *p* < 0.01. **b** Up-regulation of TFPI2 in MDA-MB-453 cells transfected with pcDNA 3.1-TFPI2 detected by western blot. **c** Over-expression of TFPI2 inhibited cell viability of MDA-MB-453 cells. ** represents pcDNA 3.1-TFPI2 vs. pcDNA 3.1-NC, *p* < 0.01. **d** Over-expression of TFPI2 inhibited cell proliferation of MDA-MB-453 cells. * represents pcDNA 3.1-TFPI2 vs. pcDNA 3.1-NC, *p* < 0.05

**Fig. 3:**
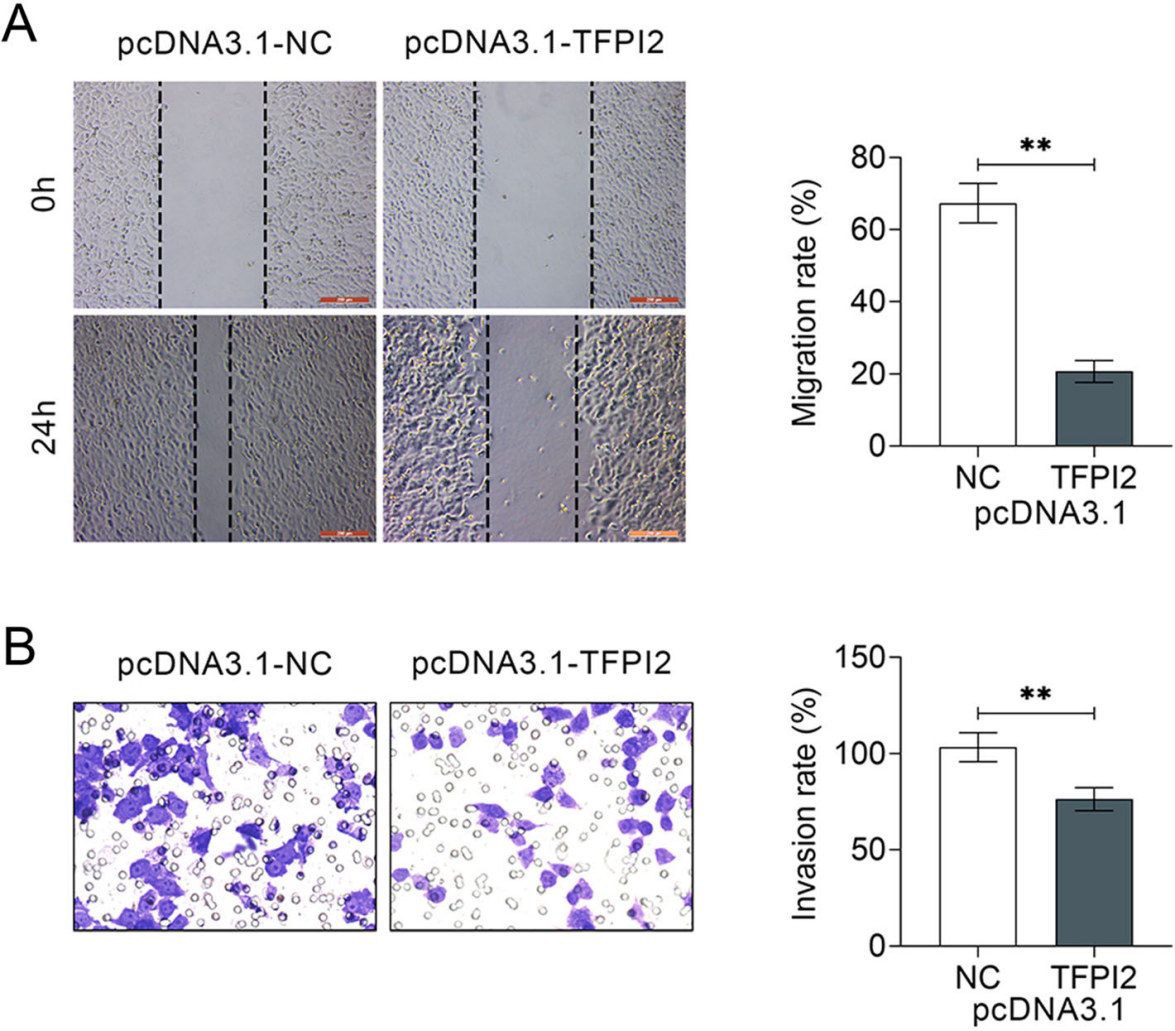
Over-expression of TFPI2 suppressed migration and invasion of breast cancer. **a** Over-expression of TFPI2 inhibited cell migration of MDA-MB-453 cells. ** represents pcDNA 3.1-TFPI2 vs. pcDNA 3.1-NC, *p* < 0.01. **b** Over-expression of TFPI2 inhibited cell invasion of MDA-MB-453 cells. ** represents pcDNA 3.1-TFPI2 vs. pcDNA 3.1-NC, *p* < 0.01

Gain-of functional assays were conducted via transfection of pcDNA 3.1-TFPI2 into MDA-MB-453 cell to determine the effects of TFPI2 on breast cancer proliferation. Over-expression efficiency was confirmed in mRNA (Fig. 2a) and protein (Fig. 2b) levels. CCK8 (Fig. 2c) and colony formation assay (Fig. 2d) results indicated that over-expression of TFPI2 deceased cell viability and inhibited cell proliferation of MDA-MB-453 cells, suggesting the anti-proliferative ability of TFPI2 on breast cancer. Wound healing (Fig. 3a) and transwell (Fig. 3b) assays showed that over-expression of TFPI2 suppressed cell migration and invasion of MDA-MB-453 cells, suggesting the anti-invasive ability of TFPI2 on breast cancer. MCF10A cells were also transfected with pcDNA 3.1-TFPI2 (Supplemental Figure S1B). Over-expression of TFPI2 significantly decreased cell viability (Supplemental Figure S1C), while had no significant effect on cell proliferation (Supplemental Figure S1D). Moreover, cell migration (Supplemental Figure S1E) and invasion (Supplemental Figure S1F) were also not significantly affected by TFPI2 over-expression.

### TFPI2 regulated TWIST1 expression in breast cancer

**Fig. 4:**
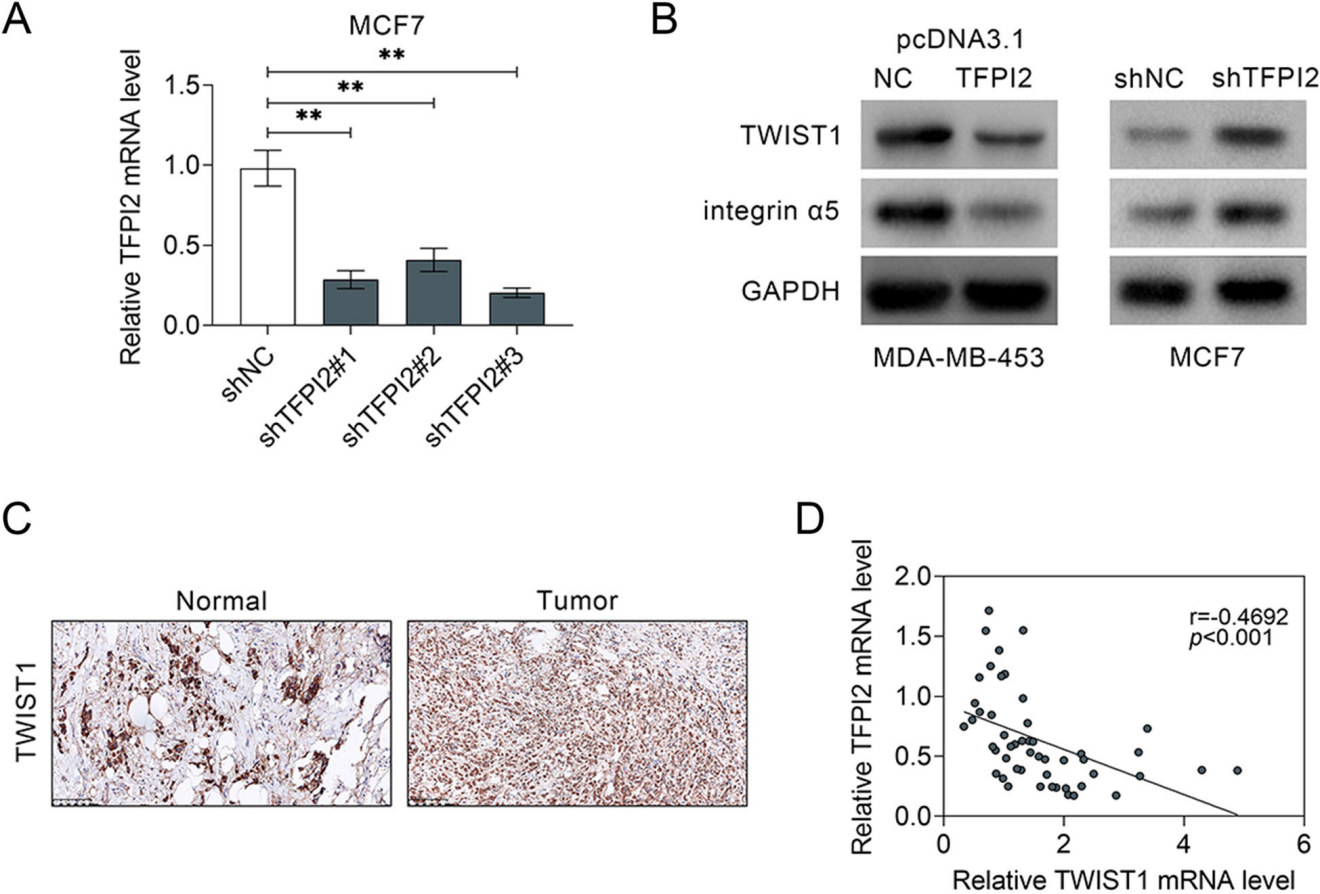
TFPI2 regulated TWIST1 expression in breast cancer. **a** Down-regulation of TFPI2 in MCF7 cells transfected with shTFPI2#1. #2, #3 indicated by qRT-PCR. ** represents shTFPI2#1. #2, #3 vs. shNC, *p* < 0.01. **b** Over-expression of TFPI2 inhibited protein expression of TWIST1 and integrin α5, while inhibition of TFPI2 promoted TWIST1 and integrin α5. **c** Immunohistochemistry of TWIST1 in Normal and Tumor breast cancer tissues, suggesting up-regulation of TWIST1 in tumor tissues. **d** Negative correlation TFPI2 and TWIST1 in breast cancer patients

Since TWIST1 has been reported to be involved in progression of breast cancer, the effect of TFPI2 on TWIST1 expression was determined in breast cancer. Knockdown of TFPI2 in MCF7 cells via shRNAs transfection was demonstrated in Fig. 4a. In addition, transfection with shRNAs in CCD-1095Sk, MDA-MB-453 and MDA-MB-468 cells also reduced the mRNA expression of TFPI2 (Supplemental Figure S1A). Meanwhile, shTFPI2#3 with the lowest expression of TFPI2 was selected for the following loss-of functional assays and named as shTFPI2. Western blot analysis indicated that TWIST1 was decreased in MDA-MB-453 cells transfected with pcDNA-3.1 TFPI2, while increased in MCF7 cells transfected with shTFPI2 (Fig. 4b), suggesting the negative correlation between TFPI2 and TWIST1 in breast cancer. Moreover, integrin α5 is the downstream target of TWIST1 which can regulate invasion of tumors (Nam et al. 2015). Consistent with expression of TWIST1 in MDA-MB-453 cells, the expression of integrin α5 was also decreased by pcDNA-3.1 TFPI2 and up-regulated by shTFPI2 (Fig. 4b), suggesting TWIST1/integrin α5 axis in regulation of breast cancer progression. Immunohistochemistry results showed up-regulation of TWIST1 in breast cancer tissues (Fig. 4c), and a significantly negative correlation between TFPI2 and TWIST1 in breast cancer was determined via bivariate correlation analysis (Fig. 4d).

### Inhibition ability of TFPI2 on breast cancer progression was reversed by TWIST1 over-expression

**Fig. 5:**
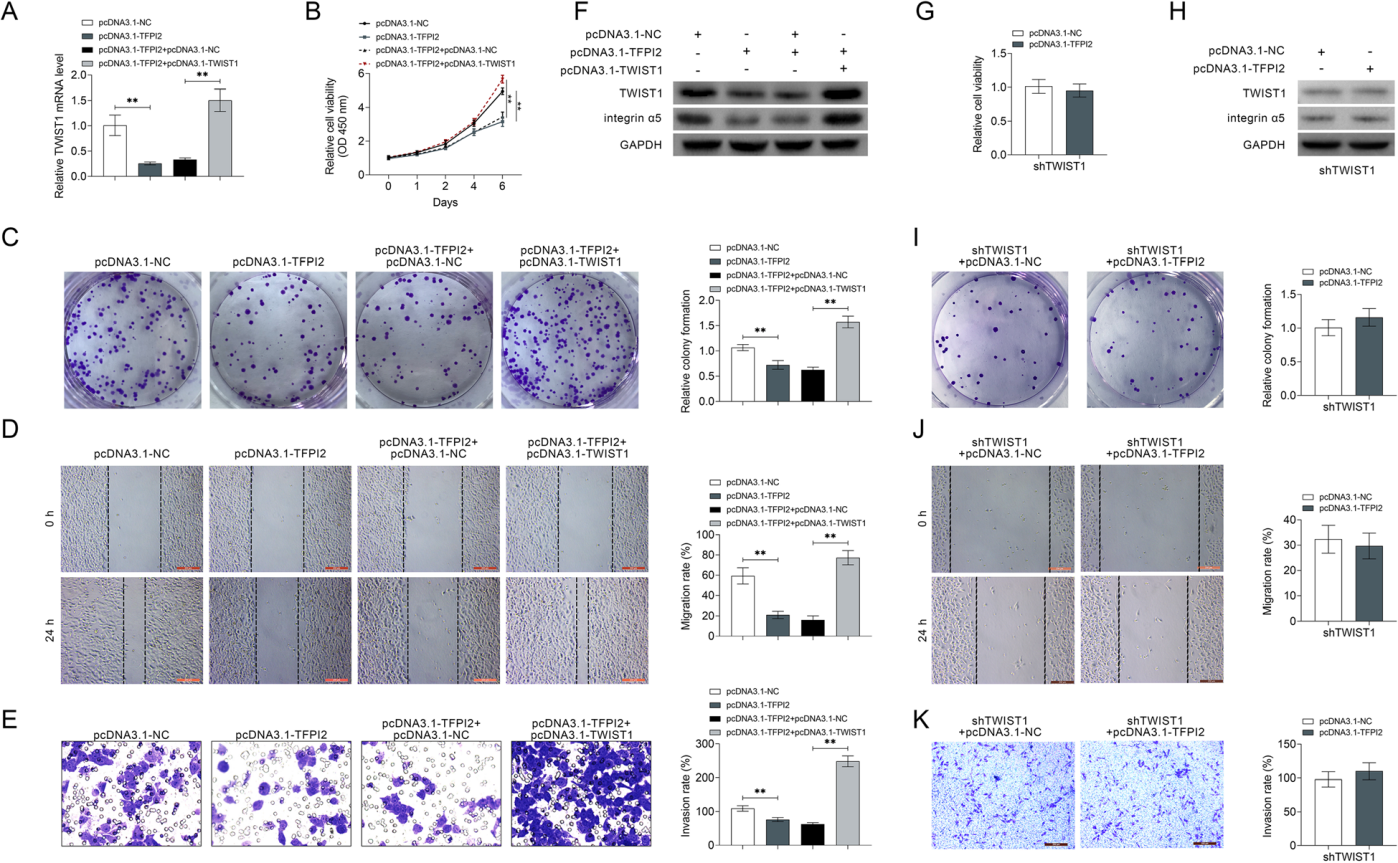
Inhibition ability of TFPI2 on breast cancer progression was reversed by TWIST1 over-expression. **a** The mRNA expression of TWIST1 was decreased in cells transfected with pcDNA 3.1-TFPI2, while reversed in cells cotransfected with pcDNA 3.1-TFPI2 and pcDNA 3.1-TWIST1. ** represents pcDNA 3.1-TFPI2 vs. pcDNA 3.1-NC or pcDNA 3.1-TFPI2 + pcDNA 3.1-TWIST1 vs. pcDNA 3.1-TFPI2 + pcDNA 3.1-NC, *p* < 0.01. **b** pcDNA 3.1-TFPI2 inhibited cell viability of MDA-MB-453 cells, while cotransfection with pcDNA 3.1-TFPI2 and pcDNA 3.1-TWIST1 promoted the cell viability. ** represents pcDNA 3.1-TFPI2 vs. pcDNA 3.1-NC or pcDNA 3.1-TFPI2 + pcDNA 3.1-TWIST1 vs. pcDNA 3.1-TFPI2 + pcDNA 3.1-NC, *p* < 0.01. **c** Transfection with pcDNA 3.1-TFPI2 inhibited cell proliferation of MDA-MB-453 cells, while cotransfection with pcDNA 3.1-TFPI2 and pcDNA 3.1-TWIST1 promoted the cell proliferation. ** represents pcDNA 3.1-TFPI2 vs. pcDNA 3.1-NC or pcDNA 3.1-TFPI2 + pcDNA 3.1-TWIST1 vs. pcDNA 3.1-TFPI2 + pcDNA 3.1-NC, *p* < 0.01. **d** Transfection with pcDNA 3.1-TFPI2 inhibited cell migration of MDA-MB-453 cells, while cotransfection with pcDNA 3.1-TFPI2 and pcDNA 3.1-TWIST1 promoted the cell migration. ** represents pcDNA 3.1-TFPI2 vs. pcDNA 3.1-NC or pcDNA 3.1-TFPI2 + pcDNA 3.1-TWIST1 vs. pcDNA 3.1-TFPI2 + pcDNA 3.1-NC, *p* < 0.01. **e** Transfection with pcDNA 3.1-TFPI2 inhibited cell invasion of MDA-MB-453 cells, while cotransfection with pcDNA 3.1-TFPI2 and pcDNA 3.1-TWIST1 promoted the cell invasion.. ** represents pcDNA 3.1-TFPI2 vs. pcDNA 3.1-NC or pcDNA 3.1-TFPI2 + pcDNA 3.1-TWIST1 vs. pcDNA 3.1-TFPI2 + pcDNA 3.1-NC, *p* < 0.01. **f** Protein expression of TWIST1 and integrin α5 were down-regulated in cells transfected with pcDNA 3.1-TFPI2, while up-regulated in cells cotransfected with pcDNA 3.1-TFPI2 and pcDNA 3.1-TWIST1. **g** Cotransfection with pcDNA 3.1-TFPI2 and shTWIST1 had no significant effect on cell viability of MCF7 cells compared to cells cotransfected pcDNA 3.1-NC with shTWIST1. **h** Cotransfection with pcDNA 3.1-TFPI2 and shTWIST1 had no significant effect on protein expression of TWIST1 and integrin α5 in MCF7 cells compared to cells cotransfected pcDNA 3.1-NC with shTWIST1. **i** Cotransfection with pcDNA 3.1-TFPI2 and shTWIST1 had no significant effect on cell proliferation of MCF7 cells compared to cells cotransfected pcDNA 3.1-NC with shTWIST1. **j** Cotransfection with pcDNA 3.1-TFPI2 and shTWIST1 had no significant effect on cell migration of MCF7 cells compared to cells cotransfected pcDNA 3.1-NC with shTWIST1. **k** Cotransfection with pcDNA 3.1-TFPI2 and shTWIST1 had no significant effect on cell invasion of MCF7 cells compared to cells cotransfected pcDNA 3.1-NC with shTWIST1

To establish the effect of TFPI2/TWIST1 axis on breast cancer progression, MDA-MB-453 cells were cotransfected with pcDNA 3.1-TFPI2 and pcDNA 3.1-TWIST1. mRNA expression of TWIST1 was decreased in cells transfected with pcDNA 3.1-TFPI2, while reversed in cells cotransfected with pcDNA 3.1-TFPI2 and pcDNA 3.1-TWIST1 (Fig. 5a). CCK8 (Fig. 5b) and colony formation (Fig. 5c) assays showed that the inhibition ability of TFPI2 on breast cancer cell proliferation was promoted by additional transfection of pcDNA 3.1-TWIST1. Moreover, results from cell migration (Fig. 5d) and invasion (Fig. 5e) analysis indicated that the suppression abilities of TFPI2 on cell migration and invasion were restored in cells cotransfected with pcDNA 3.1-TFPI2 and pcDNA 3.1-TWIST1. All these results revealed that the inhibition effect of TFPI2 on breast cancer progression was reversed by TWIST1 over-expression, which confirmed the effect of TFPI2/TWIST1 axis on regulation of breast cancer progression. The down-regulation of TWIST1 protein expression by TFPI2 over-expression was also reversed by additional over-expression of TWIST1 (Fig. 5f). Moreover, MCF7 cells were also cotransfected with pcDNA 3.1-TFPI2 and shTWIST1. However, cotransfection with pcDNA 3.1-TFPI2 and shTWIST1 had no significant effect on cell viability (Fig. 5g), protein expression of TWIST1 and integrin α5 (Fig. 5h), cell proliferation (Fig. 5i), cell migration (Fig. 5j) and invasion (Fig. 5k) compared to cells cotransfected pcDNA 3.1-NC with shTWIST1.

### TFPI2 over-expression inhibited in vivo breast cancer tumor growth

**Fig. 6:**
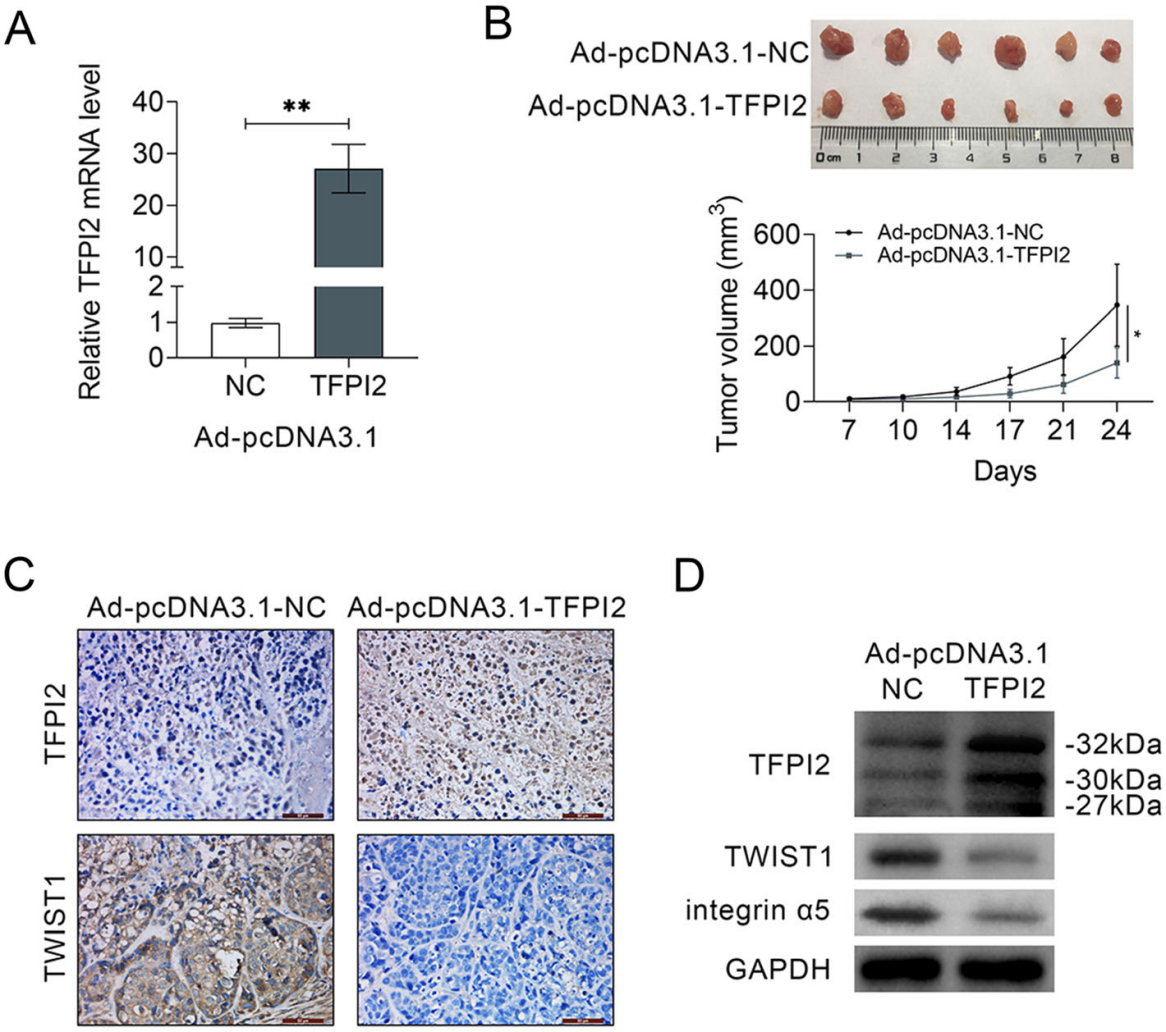
TFPI2 over-expression inhibited in vivo breast cancer tumor growth. **a** Transfection efficiency of Ad-pcDNA 3.1-TFPI2 in nude mice, suggesting up-regulation in mice injected with Ad-pcDNA 3.1-TFPI2. ** represents Ad-pcDNA 3.1-TFPI2 vs. Ad-pcDNA 3.1-NC, *p* < 0.01. **b** Injection of Ad-pcDNA 3.1-TFPI2 inhibited breast cancer tumor growth and volume. * represents Ad-pcDNA 3.1-TFPI2 vs. Ad-pcDNA 3.1-NC, *p* < 0.05. **c** Immunohistochemistry analysis of TFPI2 and TWIST1 in tissues of mice intratumoral injected with Ad-pcDNA 3.1-TFPI2 or Ad-pcDNA 3.1-NC, suggesting up-regulation of TFPI2 and down-regulation of TWIST1 in mice injected with Ad-pcDNA 3.1-TFPI2. **d** Injection of Ad-pcDNA 3.1-TFPI2 promoted on proteins expression of TFPI2, while inhibited TWIST1 and integrin α5 in xenograft tumor mice

The in vivo xenograft model was conducted via inoculation of Ad-pcDNA 3.1-TFPI2 into nude mice to investigate clinical application of TFPI2 in breast cancer. Elevation of TFPI2 via Ad-pcDNA 3.1-TFPI2 was confirmed in Fig. 6a. Moreover, intratumoral injection of Ad-pcDNA 3.1-TFPI2 inhibited tumor growth and tumor volume (Fig. 6b). Furthermore, the proteins expression of TFPI2 was increased; while the expression of TWIST1 was decreased by Ad-pcDNA 3.1-TFPI2 indicated by immunohistochemistry (Fig. 6c) and western blot (Fig. 6d). Integrin α5 was also down-regulated in mice injected with Ad-pcDNA 3.1-TFPI2 (Fig. 6d). These results revealed that TFPI2 over-expression inhibited breast cancer growth via down-regulation of TWIST1 and integrin α5.

## Discussion

Serine proteases catalyze hydrolytic reactions to participate in important physiological processes, including angiogenesis, apoptosis, wound healing (Jang et al. 2008). Dysregulated serine proteases activities have been implicated in the pathogenesis of various cancers (Lam and Schmidt 2010). Serine protease inhibitors strictly regulate the activity of serine proteases to maintain homeostasis (Rawlings et al. 2004), and have been considered as important cancer biomarkers (Putnam et al. 2003). Kunitz serine protease inhibitors, belonging to canonical serine proteases inhibitors (Krowarsch et al. 2003), are closely involved in tumor progression (Wu et al. 2017; Li et al. 2009; Kobayashi et al. 2000; Liu et al. 2018). As a common Kunitz serine protease inhibitors TFPI2 is involved in various tumor progression, especially in breast cancer. The present study for the first time indicated that TFPI2 suppressed breast cancer progression through inhibiting TWIST-integrin α5 pathway.

In consistent with previous studies (Xu et al. 2013; Guo et al. 2007), TFPI2 was down-regulated in breast cancer tissues. Low expression of TFPI2 was significantly related to tumor size, metastasis and pathological stage of breast cancer patients, which was in line with Cheng et al (Xu et al. 2013). They reported that TFPI2 was related to poor prognosis of breast cancer, suggesting the potential of TFPI2 as a prognostic biomarker for breast cancer. Moreover, in vitro functional assays revealed that over-expression of TFPI2 inhibited cell viability, proliferation, migration and invasion of breast cancer cells, confirming the clinical results of TFPI2 in breast cancer. In vivo subcutaneous xenotransplanted tumor model also revealed that ectopic expression of TFPI2 could suppress in vivo tumorigenic ability of breast cancer. In conclusion, TFPI2 might function as potential prognostic biomarker for breast cancer, and a potential novel target for the breast cancer therapy.

Although TFPI2 has been implicated in progression of various tumors, the exact regulatory mechanisms remain unclear. Epigenetic inactivation via hypermethylation of promoter CpG island of TFPI2 is associated with TFPI2silencing in pancreatic ductal adenocarcinoma (Sato et al. 2005), gastric cancer (Takada et al. 2010), non-small-cell lung cancer (Rollin et al. 2005) and glioblastomas (Vaitkiene et al. 2012), and contributes to the aggressive phenotype of various tumors. More recently, CpG methylation in the TFPI2promoter was also found in breast cancer (Guo et al. 2007). However, the mechanism linking TFPI-2 to breast cancer progression remains unknown. TWIST1 is widely known as EMT-inducing transcription factor that promote cell migration and invasion, and is also shown to be prognostic biomarker for breast cancer (Imani et al. 2016). Knockout of TWIST1 inhibited breast cancer metastasis (Xu et al. 2017b). Therefore, the regulatory mechanism of TFPI2 in breast cancer progression might be associated with TWIST1 expression. The present study showed that TWIST1 was negatively correlated with TFPI2 in breast cancer patients, and the inhibition ability of TFPI2 on breast cancer progression was reversed by over-expression of TWIST1. The effect of TFPI2 on EMT in breast cancer needs to be further investigated.

The underlying mechanism involved in regulation of breast cancer progression via TFPI2-mediated TWIST1 was then determined. Signaling pathways involved in EMT transition, such as Wnt, participated in regulation of TWIST1 on breast cancer (Glackin 2014). In addition, energy metabolism reprogramming pathways, PI3K/AKT and p53, were also involved in promotion of breast cancer via TWIST1 (Yang et al. 2015). Down-regulation of luminal breast cancer marker (Foxa1) and up-regulation of basal-like breast cancer markers (integrin α5 and integrin β1) were regulated by TWIST1 to promote breast cancer progression (Xu et al. 2017a). Our results showed that TFPI2-mediated down-regulation of TWIST1 decreased integrin α5 thereby inhibiting breast cancer progression. However, since integrin α5 and integrin β1 served as important mesenchymal marker to regulate EMT during breast cancer (Nam et al. 2015), the effect of TFPI2-mediated TWIST1 on regulation of integrin β1 also needs to be further investigated. Moreover, focal adhesion kinase (FAK) signaling pathway was involved in integrin α5 (Huang et al. 2016) and integrin β1 (31)-mediated breast cancer metastasis, and integrin α5 and integrin β1 were related to PI3K/Akt signaling (Watabe et al. 2011). The downstream signaling pathway involved in TFPI2/TWIST1/integrin α5 axis in breast cancer progression should also be investigated in the future study.

## Conclusion

TFPI2 inhibited TWIST1 expression to down-regulate integrin α5, and TFPI2/TWIST1/integrin α5 axis contributed to suppression of breast cancer progression, suggesting a novel insight into the treatment of the disease.

## Supplementary information

**Additional file 1: Figure S1.** Effect of TFPI2 on cell progression of MCF10A cells. (A) Down-regulation of TFPI2 in CCD-1095Sk, MDA-MB-453 and MDA-MB-468 cells transfected with shTFPI2#1. #2, #3 by qRT-PCR. ** represents shTFPI2#1. #2, #3 vs. shNC, *p* < 0.01. (B) Up-regulation of TFPI2 in MCF10A cells transfected with pcDNA 3.1-TFPI2 by western blot. (C) Over-expression of TFPI2 inhibited cell viability of MCF10A cells. * represents pcDNA 3.1-TFPI2 vs. pcDNA 3.1-NC, *p* < 0.05. (D) Over-expression of TFPI2 had no significant effect on cell proliferation of MCF10A cells. (E) Over-expression of TFPI2 had no significant effect on cell migration of MCF10A cells. (F) Over-expression of TFPI2 had no significant effect on cell invasion of MCF10A cells.

## Data Availability

All data generated or analyzed during this study are included in this published article.

## Abbreviations

TFPI2: Tissue factor pathway inhibitor 2
qRT-PCR: quantitative real-time polymerase chain reaction
CCK8: Cell Counting Kit-8

## References

[CR1] Albuquerque AP, Balmana M, Reis CA, Beltrão EI (2018). Identification of appropriate housekeeping genes for quantitative RT-PCR analysis in MDA-MB-231 and NCI-H460 human cancer cell lines under hypoxia and serum deprivation. J Mol Clin Med..

[CR2] Benson JR, Jatoi I, Keisch M, Esteva FJ, Makris A, Jordan VC (2009). Early breast cancer. Lancet..

[CR3] Chand HS, Schmidt AE, Bajaj SP, Kisiel W (2004). Structure-function analysis of the reactive site in the first Kunitz-type domain of human tissue factor pathway inhibitor-2. J Biol Chem.

[CR4] Feng Y, Wang Y, Zhao X, Mu Y, Lv S, Li Y (2018). Correlation between the intrinsic subtype and diagnostic methods of axillary lymph node metastasis in primary breast cancer. Eur J Gynaecol Oncol.

[CR5] Glackin CA (2014). Targeting the Twist and Wnt signaling pathways in metastatic breast cancer. Maturitas..

[CR6] Guo H, Lin Y, Zhang H, Liu J, Zhang N, Li Y (2007). Tissue factor pathway inhibitor-2 was repressed by CpG hypermethylation through inhibition of KLF6 binding in highly invasive breast cancer cells. BMC Mol Biol.

[CR7] Hashim D, Boffetta P, La Vecchia C, Rota M, Bertuccio P, Malvezzi M (2016). The global decrease in cancer mortality: trends and disparities. Ann Oncol.

[CR8] Herman MP, Sukhova GK, Kisiel W, Foster D, Kehry MR, Libby P (2001). Tissue factor pathway inhibitor-2 is a novel inhibitor of matrix metalloproteinases with implications for atherosclerosis. J Clin Invest.

[CR9] Huang C, Verhulst S, Shen Y, Bu Y, Cao Y, He Y (2016). AKR1B10 promotes breast cancer metastasis through integrin alpha5/delta-catenin mediated FAK/Src/Rac1 signaling pathway. Oncotarget..

[CR10] Imani S, Hosseinifard H, Cheng J, Wei C, Fu J (2016). Prognostic value of EMT-inducing transcription factors (EMT-TFs) in metastatic breast Cancer: a systematic review and meta-analysis. Sci Rep.

[CR11] Jang IH, Nam HJ, Lee WJ (2008). CLIP-domain serine proteases in Drosophila innate immunity. BMB Rep.

[CR12] Khan MA, Chen HC, Zhang D, Fu J (2013). Twist: a molecular target in cancer therapeutics. Tumour Biol.

[CR13] Kobayashi H, Hirashima Y, Sun GW, Ohi H, Fujie M, Terao T (2000). Identification and characterization of a Kunitz-type protease inhibitor in ascites fluid from patients with ovarian carcinoma. Int J Cancer.

[CR14] Konduri SD, Tasiou A, Chandrasekar N, Rao JS (2001). Overexpression of tissue factor pathway inhibitor-2 (TFPI-2), decreases the invasiveness of prostate cancer cells in vitro. Int J Oncol.

[CR15] Krowarsch D, Cierpicki T, Jelen F, Otlewski J (2003). Canonical protein inhibitors of serine proteases. Cell Mol Life Sci.

[CR16] Lam DK, Schmidt BL (2010). Serine proteases and protease-activated receptor 2-dependent allodynia: a novel cancer pain pathway. Pain..

[CR17] Lee JJ, Chu E (2018). The adjuvant treatment of stage III Colon Cancer: might less be more?. Oncology..

[CR18] Li W, Wang BE, Moran P, Lipari T, Ganesan R, Corpuz R (2009). Pegylated kunitz domain inhibitor suppresses hepsin-mediated invasive tumor growth and metastasis. Cancer Res.

[CR19] Liu CL, Yang PS, Chien MN, Chang YC, Lin CH, Cheng SP (2018). Expression of serine peptidase inhibitor Kunitz type 1 in differentiated thyroid cancer. Histochem Cell Biol.

[CR20] Nam EH, Lee Y, Moon B, Lee JW, Kim S (2015). Twist1 and AP-1 cooperatively upregulate integrin alpha5 expression to induce invasion and the epithelial-mesenchymal transition. Carcinogenesis..

[CR21] Putnam JB, Royston D, Chambers AF, Dunbar S, Lemmer JH, Norman P (2003). Evaluating the role of serine protease inhibition in the management of tumor micrometastases. Oncology..

[CR22] Rawlings ND, Tolle DP, Barrett AJ (2004). Evolutionary families of peptidase inhibitors. Biochem J.

[CR23] Rollin J, Iochmann S, Blechet C, Hube F, Regina S, Guyetant S (2005). Expression and methylation status of tissue factor pathway inhibitor-2 gene in non-small-cell lung cancer. Br J Cancer.

[CR24] Sato N, Parker AR, Fukushima N, Miyagi Y, Iacobuzio-Donahue CA, Eshleman JR (2005). Epigenetic inactivation of TFPI-2 as a common mechanism associated with growth and invasion of pancreatic ductal adenocarcinoma. Oncogene..

[CR25] Siegel RL, Miller KD, Jemal A (2016). Cancer statistics, 2016. CA Cancer J Clin.

[CR26] Takada H, Wakabayashi N, Dohi O, Yasui K, Sakakura C, Mitsufuji S (2010). Tissue factor pathway inhibitor 2 (TFPI2) is frequently silenced by aberrant promoter hypermethylation in gastric cancer. Cancer Genet Cytogenet.

[CR27] Vaitkiene P, Skiriute D, Skauminas K, Tamasauskas A (2012). Associations between TFPI-2 methylation and poor prognosis in glioblastomas. Medicina..

[CR28] Watabe H, Furuhama T, Tani-Ishii N, Mikuni-Takagaki Y (2011). Mechanotransduction activates alpha (5) beta (1) integrin and PI3K/Akt signaling pathways in mandibular osteoblasts. Exp Cell Res.

[CR29] Wu SR, Teng CH, Tu YT, Ko CJ, Cheng TS, Lan SW (2017). The Kunitz domain I of hepatocyte growth factor activator Inhibitor-2 inhibits Matriptase activity and invasive ability of human prostate Cancer cells. Sci Rep.

[CR30] Xu C, Wang H, He H, Zheng F, Chen Y, Zhang J (2013). Low expression of TFPI-2 associated with poor survival outcome in patients with breast cancer. BMC Cancer.

[CR31] Xu Y, Lee DK, Feng Z, Xu Y, Bu W, Li Y (2017). Breast tumor cell-specific knockout of Twist1 inhibits cancer cell plasticity, dissemination, and lung metastasis in mice. Proc Natl Acad Sci U S A.

[CR32] Xu Y, Qin L, Sun T, Wu H, He T, Yang Z (2017). Twist1 promotes breast cancer invasion and metastasis by silencing Foxa1 expression. Oncogene..

[CR33] Yang L, Hou Y, Yuan J, Tang S, Zhang H, Zhu Q (2015). Twist promotes reprogramming of glucose metabolism in breast cancer cells through PI3K/AKT and p53 signaling pathways. Oncotarget..

