# Supplementary figures and images for: TFPI2 suppresses breast cancer progression through inhibiting TWIST-integrin α5 pathway
Source: Mol Med. 2020 Apr 5;26:27. doi: 10.1186/s10020-020-00158-2 (PMC7133004; doi:10.1186/s10020-020-00158-2)

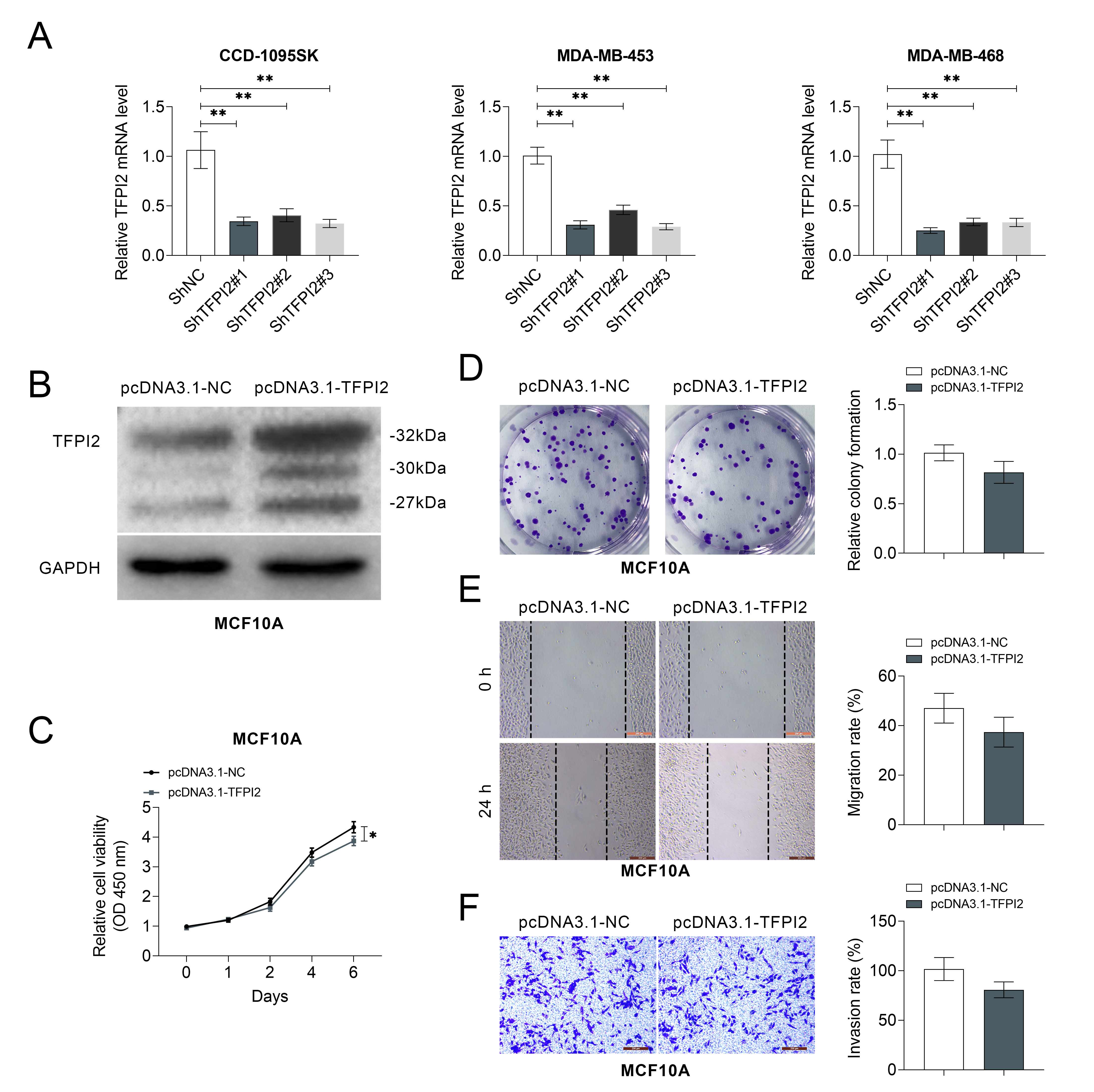

Supplement: Supplementary file 1 — Additional file 1: Figure S1. Effect of TFPI2 on cell progression of MCF10A cells. (A) Down-regulation of TFPI2 in CCD-1095Sk, MDA-MB-453 and MDA-MB-468 cells transfected with shTFPI2#1. #2, #3 by qRT-PCR. ** represents shTFPI2#1. #2, #3 vs. shNC, p < 0.01. (B) Up-regulation of TFPI2 in MCF10A cells transfected with pcDNA 3.1-TFPI2 by western blot. (C) Over-expression of TFPI2 inhibited cell viability of MCF10A cells. * represents pcDNA 3.1-TFPI2 vs. pcDNA 3.1-NC, p < 0.05. (D) Over-expression of TFPI2 had no significant effect on cell proliferation of MCF10A cells. (E) Over-expression of TFPI2 had no significant effect on cell migration of MCF10A cells. (F) Over-expression of TFPI2 had no significant effect on cell invasion of MCF10A cells. [file 10020_2020_158_MOESM1_ESM.jpg]
